# Transcriptional Regulation of Δ6-Desaturase by Peroxisome Proliferative-Activated Receptor **δ** Agonist in Human Pancreatic Cancer Cells: Role of MEK/ERK1/2 Pathway

**DOI:** 10.1155/2013/607524

**Published:** 2013-10-30

**Authors:** Maryam Darabi, Shima Byagowi, Shabnam Fayezi, Masoud Darabi, Shahab Mirshahvaladi, Mehdi Sahmani

**Affiliations:** ^1^Drug Applied Research Center, Tabriz University of Medical Sciences, Tabriz 5165665811, Iran; ^2^Department of Clinical Biochemistry and Genetics, Cellular and Molecular Research Center, Faculty of Medicine, Qazvin University of Medical Sciences, Qazvin 3411975981, Iran; ^3^Department of Anatomy and Cell Biology, Shahid Beheshti University of Medical Sciences, Tehran 193954719, Iran; ^4^Department of Biotechnology, Cellular and Molecular and Burns Research Centers, Iran University of Medical Sciences, Tehran 141556183, Iran

## Abstract

The Δ6-desaturase (Δ6D), also known as fatty acid desaturase 2, is a regulatory enzyme in *de novo* fatty acid synthesis, which has been linked to obesity and diabetes. The aim of the present study was to investigate the effect of peroxisome proliferative-activated receptor **δ** (PPAR**δ**) agonist and MEK/ERK1/2-dependent pathway on the expression of Δ6D in human pancreatic carcinoma cell line PANC-1. PANC-1 cells cultured in RPMI-1640 were exposed to the commonly used ERK1/2 pathway inhibitor PD98059 and PPAR**δ** agonist GW0742. Changes in mRNA and protein expression of Δ6D were then determined using real-time RT-PCR and Western blot, respectively. The expression of Δ6D (*P* < 0.01) increased following treatment with PPAR**δ** agonist both at mRNA and protein levels, whereas no significant change was observed after treatment with MEK/ERK1/2 pathway inhibitor. It was also found that the increase in the expression of Δ6D in response to GW0742 was significantly inhibited by PD98059 (>40%, *P* < 0.05) or EGF receptor-selective inhibitor AG1478 (>25%, *P* < 0.05) pretreatment. PPAR**δ** and MEK/ERK1/2 signaling pathways affect differentially the expression of Δ6D in pancreatic cancer cells. Furthermore, there may be an inhibitory crosstalk between these two regulatory pathways on the mRNA expression of Δ6D and subsequently on Δ6D protein expression.

## 1. Introduction

Numerous *in vitro* and *in vivo* studies indicate the critical role of fatty acids in cell membrane fluidity, which in turn affect ligand binding and cellular signal transduction of surface receptors and G-proteins [1–3]. This role has been demonstrated by the fact that the altered levels of fatty acid desaturase enzymes are associated with various human diseases like diabetes and atherosclerosis [4, 5]. Studies have shown that lipotoxicity of human pancreatic islets, which is attributed to accumulation of saturated fatty acids, is one of the important causes of dysregulated insulin secretion and apoptosis of pancreatic *β*-cell [6, 7]. In contrast to saturated fatty acids, unsaturated fatty acids play a key role in survival of the pancreatic *β*-cell [8, 9]. The membrane-bound enzyme Δ6 fatty acid desaturase (Δ6D), encoded by the *fatty acid desaturase 2* (*FADS2*) gene, is the first and rate-limiting enzyme in the synthesis of unsaturated fatty acids. *FADS2*-deficient mouse model has revealed that Δ6D is the main enzyme in *in vivo* production of n-6 polyunsaturated fatty acids (PUFA) [10].

The delta isoform of the peroxisome proliferator-activated receptor (PPAR) *δ* is a family of nuclear receptors regulating the expression of genes involved in fatty acid metabolism. Previous studies have reported that both PUFA and PPAR*α* agonist response elements are present in the *FADS2*; however, no exact region that responds independently to PPAR*δ* has yet been identified [11]. A high affinity synthetic PPAR*δ* agonist has been shown to modulate fatty acid metabolic pathways, particularly those involving n-6 PUFA desaturation [12]. However, the exact functional targets in these pathways have also not yet been detected.

PPAR*δ* like other nuclear receptors may be affected by other signaling pathways, and this crosstalk might modulate the activity of these kinds of receptors. We recently demonstrated that the mitogen-activated protein kinase (MAPK) MEK/ERK1/2 signaling inhibition could alter the expression level of Δ6D in hepatocellular carcinoma cell line HepG2 [13]. It has previously been shown that inhibition of ERK1/2 signaling had no apparent effect on PPAR*δ* agonist-mediated increase in glucose uptake in cultured human skeletal muscle, whereas PPAR*δ* agonist increased both phosphorylation and expression of ERK 1/2[14]. Thus, it is possible that both Erk1/2 signaling and PPAR*δ* are involved in a crosstalk contributing to the regulation of Δ6D expression.

Several lines of evidence suggest that PPAR*δ* activity and Erk1/2 signaling play important role in the regulation of pancreatic *β*-cells function. In the present study in human pancreatic carcinoma cell line PANC-1, Δ6D expression was tested for responsiveness to the synthetic PPAR*δ* agonist GW0742, under either MEK/ERK1/2 or epidermal growth factor receptor (EGFR) signaling pathway blockade.

## 2. Materials and Methods

### 2.1. Materials

Cell culture materials, media, and FBS were obtained from Sigma Chemicals Company (St. Louis, Mo, USA). GW0742 and PD98059 were purchased from Cayman Chemicals (Ann Arbor, MI, USA). PANC-1 cell line was obtained from the Pasteur Institute Culture Collection in Tehran, Iran. All other chemicals used were of analytical grade.

### 2.2. Cell Culture

PANC-1 cells were grown in RPMI1640 containing 10% FBS, L-glutamine (2 mM), penicillin (100 units/mL), and streptomycin (100 *μ*g/mL) at 37°C, 5% CO_2_/95% humidity. The subcultures with less than 8 passages were used for drug treatment experiments. The cells were seeded at a density of 2 × 10^5^/well in a 6-well plate. After allowing the cells to attach overnight, the medium was replaced with fresh medium containing ±PPAR*δ* agonist GW0742, specific inhibitor of the MEK/ERK1/2 PD98059, or selective inhibitor of EGFR AG1478. Following 48 h incubation, culture medium was removed; the cell monolayer was washed and collected for mRNA and protein expression analysis.

### 2.3. Real-Time RT-PCR Analysis

Total RNAs were purified with QIAamp RNA mini kit with a DNase I treatment (Qiagen GmbH, Hilden, Germany) according to the manufacturer's instructions. Total RNAs were then resuspended in 50 *μ*L of RNase-free water and stored at –80°C. For cDNA synthesis, RNA (1 *μ*g) was reverse transcribed with a first-strand cDNA synthesis kit for reverse-transcription polymerase chain reaction (RT-PCR; Roche, Hertfordshire, UK).

*FADS2* primers [15] for real-time PCR were designed to amplify a segment in the cDNA sequence as follows: forward primer TTACAACATCACCAAATGGTCCAT, the intronspanning reverse primer GAAGGCATCCGTTGCATCTT, and the labeled probe CCAGCGGGTCATCGGGCACTAC. The TaqMan probes were labeled with a reporter dye (FAM) on its 5′ end and a quencher dye (TAMRA) on its 3′ end. Glyceraldehyde-3-phosphate dehydrogenase (GAPDH) mRNA was measured using the predeveloped TaqMan Assay. Real-time PCR reactions were performed on an Applied Biosystems StepOnePlus Real-Time PCR System according to the standard protocols of the manufacturer. Samples were assayed in triplicates.

Quantification was performed according to the relative standard curve method described in the PE User bulletin no. 2. Quantity of FADS2 mRNA was divided by GAPDH mRNA content, and the normalized quantity expressed as a unitless number, and all quantities are expressed as an *x*-fold difference relative to a calibrator.

### 2.4. Western Blot Analysis

Cells were washed twice with PBS and placed in lysis buffer containing antiprotease cocktail (Roche Diagnostics, IN, USA). Protein concentration in the supernatant of lysed cells was measured using Bradford's colorimetric method with reference to BSA standards (Bio-Rad). Western blot analysis was performed according to the standard procedures (Bio-Rad, Richmond, CA, USA). Briefly, 30 *μ*g of whole cell extract was separated by SDS PAGE. After electrotransfer to Immobilon-P membrane (Millipore, Bedford, MA, USA), the blots were blocked with 3% skim milk and subjected to Western blot analysis with either polyclonal anti-Δ6D or anti-*β*-actin (Abcam, Cambridge, MA, USA). Immunoreactive bands were detected by enhanced ECL (Amersham Bioscience). For quantification, the developed films were scanned and pixel intensity of Δ6D signal was normalized against *β*-actin for each sample.

### 2.5. Statistical Analyses

Data are presented as mean ± SE. Experiments were repeated three times in duplicate. Statistically significant differences in mean values between groups were assessed by ANOVA test with post hoc Tukey's test for multiple comparisons. A *P* value < 0.05 was considered statistically significant. All analyses were carried out using SPSS for windows version 11.0 (SPSS Inc., Chicago, IL, USA).

## 3. Results

To define if there is a connection between PPAR*δ* and ERK1/2 MAPK signaling pathway on the expression of Δ6D enzyme, PANC-1 cells were treated with a specific PPAR*δ* agonist (GW0742), a selective inhibitor of MAP kinase (PD98059), or a EGF receptor-selective tyrosine kinase inhibitor (AG1478).

**Figure 1:**
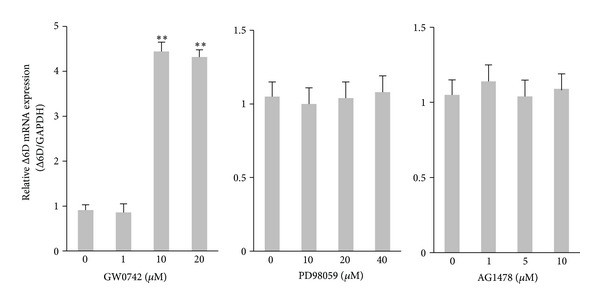
Effects of different doses of the PPAR*δ* agonist, selective inhibitor of MEK/ERK1/2, and EGF receptor-selective tyrosine kinase inhibitor on mRNA expression of Δ6-desaturase (Δ6D) in PANC-1 human pancreatic tumor cells. Cells were treated with GW0742, PD98059, or AG1478 as indicated. One microgram of extracted RNA was reverse transcribed, and real-time RT-PCR to amplify Δ6 desaturase and GAPDH cDNA fragments was performed as described. Relative expression with duplicate samples is given and was calculated by normalization to GAPDH and represented as mean ± SE from 3 independent experiments. Data are means ± SE, *n* = 3. **: *P* < 0.01 for Student's *t*-test.

To optimize the assay, cultured PANC-1 cells were incubated with different concentrations of GW0742 (0–20 *μ*M), PD98059 (0–40 *μ*M), or AG1478 (0–10 *μ*M) 48 h at 37°C (Figure 1). At 1 *μ*M concentration, GW0742 induced no apparent effect on Δ6D mRNA expression. At 10–20 *μ*M of GW0742, Δ6D expression was significantly upregulated (>4.3-fold, *P* < 0.01) in PANC-1 cells. The treatment with all three doses of PD98059 and AG1478 induced no significant changes in the mRNA expression of Δ6D compared with that of the control. 

**Figure 2:**
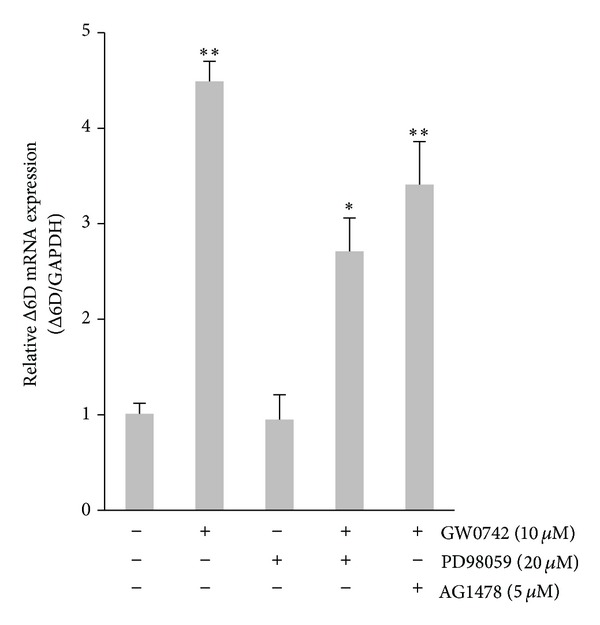
Effects of the PPAR*δ* agonist, selective inhibitor of MEK/ERK1/2, and EGF receptor-selective tyrosine kinase inhibitor on mRNA expression of Δ6-desaturase (Δ6D) in PANC-1 human pancreatic tumor cells. Cells were treated with GW0742, PD98059, or AG1478 as indicated. One microgram of extracted RNA was reverse transcribed, and real-time RT-PCR to amplify Δ6 desaturase and GAPDH cDNA fragments was performed as described. Relative expression with duplicate samples is given and was calculated by normalization to GAPDH and represented as mean ± SE from 3 independent experiments. * and **: *P* < 0.05 and *P* < 0.01 for Student's *t*-test, respectively.

**Figure 3:**
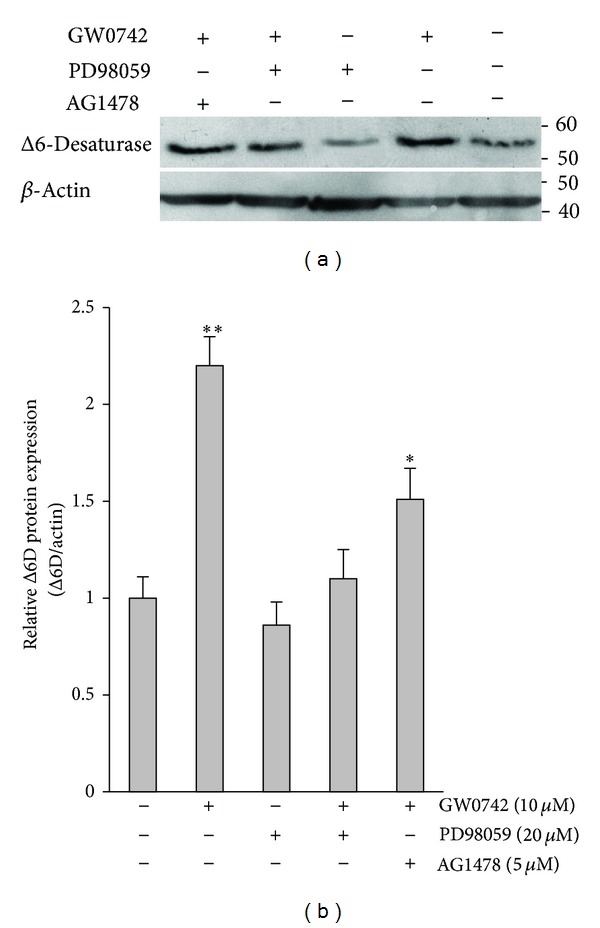
Effects of the PPAR*δ* agonist, selective inhibitor of MEK/ERK1/2, and EGF receptor-selective tyrosine kinase inhibitor on protein expression of Δ6-desaturase (Δ6D) in PANC-1 human pancreatic tumor cells. Cells were treated with GW0742, PD98059, or AG1478 as indicated. Cell lysates were analyzed for Δ6 desaturase and *β*-actin. Molecular weight markers are shown. Δ6 desaturase levels were detected by Western blots, quantified, normalized to *β*-actin, and represented as mean ± SE from 3 independent experiments. * and **: *P* < 0.05 and *P* < 0.01 for Student's *t*-test, respectively.

The selected doses were then studied on the both mRNA and protein expression of Δ6D simultaneously (Figures 2 and 3). Similarly, treatment of PANC-1 cell cultures with GW0742 significantly increased the protein expression level of Δ6D (2.2-fold, *P* < 0.01). There was also no significant effect on cellular Δ6D protein expression following PD98059 incubation (Figure 3). Comparison of control with the combined drug condition showed a significant increase only in the mRNA expression of Δ6D (2.7-fold, *P* = 0.02). It was also found that the increase in expression of Δ6D in response to the PPAR*δ* agonist was significantly inhibited (>40%, *P* = 0.032) by PD98059 pretreatment. EGF receptor-selective inhibitor AG1478 also significantly reduced stimulatory effect of GW0742 on mRNA (26%, *P* = 0.032) and protein (32%, *P* = 0.032) expression of Δ6D. However, the inhibitory effect of AG1478 at the protein level was very modest when compared to the PD98059. 

## 4. Discussion

Regulation of carbohydrate and lipid metabolism in response to the glucose consumption is mediated by insulin secretion from pancreatic *β*-cells [16]. Several previous studies in pancreatic islets and glucose-induced beta-(INS-1)-cell have shown that fatty acids could modulate insulin resistance [17–19] by inducing a number of unique responses such as resistance to cytokine-induced *β*-cell destruction, altered insulin gene expression, and controlling proinflammatory mediators derived from n-6 PUFAs [20]. In a previous study, we suggested that Δ6D may act as potential mediator of the effects of ERK1/2 signaling on hepatic fatty acid composition [13]. Krämer et al.[14] have reported that PPAR*δ* agonists could increase phosphorylation and expression of MAPK ERK1/2 by 2.2-fold. According to this evidence, it seems that MAPK cascade signaling could be modulated by PPAR*δ* activity.

In this study, it was demonstrated that PPAR*δ* agonist GW0742 could markedly increase Δ6D gene and protein expression in pancreatic carcinoma cell line PANC-1. Accordingly, Roberts et al. [12] have shown that PPAR*δ* agonist could increase unsaturated fatty acid products of the Δ6D in liver, skeletal muscle, blood serum, and white adipose tissue from obese mice. These results confirm an earlier study which has shown that *FADS2 *may contain peroxisome proliferator response elements which are under positive control of peroxisome proliferators [11]. 

In the study by Cohen [21] and colleagues, it has been shown that PPAR*δ* and PUFA play a key role in insulin secretion from isolated islet cells and INS-1E. According to this study, high glucose level in islet cell could increase the release of n-6 PUFA, which acts as endogenous ligand for PPAR*δ*. According to our present findings, it is thus possible that the PPAR*δ*-mediated general response of *β*-cell to increased glucose level is coupled to the production of unsaturated fatty acids via enzymatic Δ6 desaturation. Taken together, these results signify the role of PPAR*δ* and Δ6 fatty acid desaturation in potency of insulin secretion from pancreatic *β*-cell.

Our findings on PANC-1 cultures incubated with MEK/ERK1/2 inhibitor PD98059 showed no apparent change in Δ6D mRNA and protein expression compared to untreated control cells. In contrast with these observations, our results in human hepatoblastoma (HepG2) cells indicated that the expression level of Δ6D was significantly increased in the presence of MEK/ERK1/2 inhibitor. The differences in our results may be due to either a cell-type-specific effect or different sensitivities in methods of measurement and use of relative RT-PCR versus real-time RT-PCR. 

We also demonstrated that treatment with both ERK1/2 inhibitor and EGFR inhibitor remarkably downregulated GW0742-induced Δ6D mRNA and protein expression. In spite of comparable levels of Δ6D mRNA expression, EGFR inhibitor had less suppressive effect on PPAR*δ* agonist-mediated induction of Δ6D protein expression than ERK1/2 inhibitor. This difference may be related to additional changes in associated downstream signaling pathways, like PI3K-Akt [22, 23]. So it could be hypothesized that there is increased protein stability or decreased protein degradation in response to inhibition of PI3K, which is downstream of EGFR. Slowed EGF-induced protein degradation following inhibition of PI3K signaling has previously been reported [24].

To the best of our knowledge, this study is the first study to examine the combined effect of PPAR*δ* agonist and ERK1/2 blockade on the gene and protein expression of fatty acid Δ6D. We used PANC-1 pancreatic tumor cells which are well-characterized human-derived cells for studying human pancreatic cells *in vitro* [25]. Based on our result, we could conclude that EGFR signaling pathways maybe involved in the suppression of Δ6D expression; however, the relationship between this pathway and PPAR*δ* remains to be investigated. 

## 5. Conclusions

Our study showed that PPAR*δ* and ERK1/2 MAPK signaling pathways affect the gene expression of Δ6D in pancreatic carcinoma cell line PANC-1. Furthermore, a possible inhibitory effect of ERK1/2 MAPK signaling on PPAR*δ* activity may serve to coordinate Δ6 desaturation of fatty acids in pancreatic cells.
